# Catweasel mice: A novel role for *Six1* in sensory patch development and a model for branchio-oto-renal syndrome

**DOI:** 10.1016/j.ydbio.2009.01.030

**Published:** 2009-04-15

**Authors:** Erika A. Bosman, Elizabeth Quint, Helmut Fuchs, Martin Hrabé de Angelis, Karen P. Steel

**Affiliations:** a The Wellcome Trust Sanger Institute, The Wellcome Trust Genome Campus, Hinxton CB10 1SA, UK; b MRC Institute of Hearing Research, Nottingham NG7 2RD, UK; c Helmholtz Zentrum München, GmbH, Ingolstädter Landstraβe 1, D-85764 Neuherberg, Germany

**Keywords:** *Six1*, Mouse, BOR, Inner ear, Sensory patch, Hair cell, Kidney, Incus, *Jag1*

## Abstract

Large-scale mouse mutagenesis initiatives have provided new mouse mutants that are useful models of human deafness and vestibular dysfunction. Catweasel is a novel *N*-ethyl-*N*-nitrosourea (ENU)-induced mutation. Heterozygous catweasel mutant mice exhibit mild headtossing associated with a posterior crista defect. We mapped the catweasel mutation to a critical region of 13 Mb on chromosome 12 containing the *Six1*, -*4* and -*6* genes. We identified a basepair substitution in exon 1 of the *Six1* gene that changes a conserved glutamic acid (E) at position 121 to a glycine (G) in the Six1 homeodomain. *Cwe/Cwe* animals lack Preyer and righting reflexes, display severe headshaking and have severely truncated cochlea and semicircular canals. *Cwe/Cwe* animals had very few hair cells in the utricle, but their ampullae and cochlea were devoid of any hair cells. *Bmp4*, *Jag1* and Sox2 expression were largely absent at early stages of sensory development and *NeuroD* expression was reduced in the developing vestibulo-acoustic ganglion. Lastly we show that *Six1* genetically interacts with *Jag1*. We propose that the catweasel phenotype is due to a hypomorphic mutation in *Six1* and that catweasel mice are a suitable model for branchio-oto-renal syndrome. In addition *Six1* has a pivotal role in early sensory patch development and may act in the same genetic pathway as *Jag1*.

## Introduction

Over 300 human syndromes have been described where deafness and/or vestibular malfunction are present (Tekin et al., 2001) and many of these syndromes have severe malformations of the hearing apparatus due to abnormal inner ear development. Large-scale ENU and other mutagenesis programmes have provided the field with new mouse mutants with deafness and/or vestibular dysfunction. This has allowed us and others to identify novel candidate genes for human deafness and some of these provide good models for human diseases (Kiernan et al., 2001, 2005; Bosman et al., 2005; Vrijens et al., 2006).

Branchio-oto-renal syndrome (OMIM 113650; http://www.ncbi.nlm.nih.gov/entrez/dispomim.cgi?id=113650) is an autosomal dominant developmental disorder of kidney and urinary tract malformations and hearing impairment (Melnick et al., 1976). In humans, mutations in *EYA1* and its interacting partners *SIX1* and *SIX5* have been identified as causing BOR syndrome (Abdelhak et al., 1997; Ruf et al., 2004; Hoskins et al., 2007). This syndrome has a wide intrafamilial variability and reduced penetrance (Kumar et al., 1999). A closely related disorder is branchio-oto (BO) syndrome, where patients suffer from branchial defects and deafness without renal abnormalities (OMIM 602588), but might be a milder variant of BOR syndrome. Deletion of the *Six1* or *Eya1* genes in mice has confirmed the important role of these genes during the development of inner ear and other organs affected in BOR syndrome. Mice carrying a hypomorphic *Eya1* mutation have inner ear and other malformations that are reminiscent of those found in patients with BOR syndrome (Johnson et al., 1999). Complete loss of *Eya1* and *Six1* leads to an arrest in inner ear development at otocyst stage due to a failure of dorso-ventral (D-V) axis determination (Xu et al., 1999; Zheng et al., 2003). In addition to this early role, *Six1* and *Eya1* are both expressed during later stages of inner ear morphogenesis mainly in the developing sensory epithelium and a role for *Eya1* during sensory patch specification has been proposed (Zheng et al., 2003; Ozaki et al., 2004; Zou et al., 2006). The role of *Six1* during later stages of inner ear morphogenesis and development of the sensory patch remains to be elucidated.

Here we describe catweasel (*Cwe*), a novel ENU-induced mutation that causes mild headbobbing in heterozygous (*Cwe*/+) mice. These mice lack the eminentium cruciatum in the posterior crista and have extra inner hair cells in the organ of Corti. Catweasel maps to a 13 Mb region of chromosome 12 and we identified a point mutation in the gene encoding the Six-type homeobox protein Six1. Homozygous (*Cwe/Cwe*) mice are viable, but have kidney defects, severe vestibular abnormalities and are deaf, due to defects in both the inner and middle ear. These mice lack most sensory hair cells, and we show that sensory patch development is affected as early as E10.5. Finally we show that *Six1* interacts genetically with the Notch ligand *Jag1*. We propose that *Six1* has a pivotal role in early sensory patch development and may act in the same genetic pathway as *Jag1*. In addition, catweasel mice are a good model to study the inner ear and kidney abnormalities found in branchio-oto-renal (BOR) syndrome.

## Materials and methods

### Mice and behavioural analysis

Animal husbandry and experiments were carried out in accordance to UK Home Office regulations and with permission of the government of Oberbayern (Germany). The catweasel mutation arose from a large-scale mutagenesis screen (Hrabé de Angelis et al., 2000). Male C3HeB/FeJ mice were injected with three doses of 80 mg/kg *N*-ethyl-*N*-nitrosourea (ENU) at weekly intervals, allowed to recover and mated with uninjected C3HeB/FeJ females. F1 offspring were screened for a variety of dominantly inherited defects including deafness and vestibular defects. A custom built click box was held above the mouse and a calibrated 20 kHz tone burst at an intensity of 90 dB SPL was delivered and the presence of an ear flick response (Preyer reflex) was recorded. Other behavioural testing (rotarod, swim test, tail suspension) was performed as described (http://www.eumorphia.org/EMPReSS/servlet/EMPReSS.Frameset). The catweasel (*Cwe*, ABE4) founder was discovered because of its mild headshaking behaviour. For all analysis, unless otherwise described, the mutants used were distinguished by their clear headbobbing behaviour, and the mice were studied on their original C3HeB/FeJ genetic background.

### Genetic mapping and genotying

*Cwe*/+ animals on a C3HeB/FeJ genetic background were outcrossed to C57BL/6JIco mice. Affected *Cwe*/+ F1 offspring were backcrossed to wild type (+/+) C3HeB/FeJ animals. The DNA from 30 backcross offspring exhibiting severe headshaking behaviour was used in a genome scan to link the behavioural trait with a chromosome. A panel of 57 markers spanning the autosomes was used to detect polymorphisms between C3HeB/FeJ and C57BL/6JIco mice (Suppl. Table 1). Fine mapping was carried out using *Cwe*/+ mutants with confirmed abnormal posterior crista (*n* = 38) with additional markers on chromosome 12 (Suppl. Table 2). To genotype catweasel mice, *Six1* exon 1 was amplified by PCR using FW 5′-CACCTGCACAAGAACGAGAG-3′ and RV 5′-TTCGACTCAGACCAGCTTCA-3′ primers and sequenced with internal primer FW2 5′-ACTTCCGCGAGCTCTACAAG-3′.

### Phenotype analysis

P21 mice were sacrificed by cervical dislocation. Scanning electron microscopy was performed as described (Bosman et al., 2005). Middle ear ossicles were dissected out in PBS and photographed. For inner ear examination heads were bisected, the brain removed and the skull was fixed in Bodian's fixative (75% ethanol, 5% acetic acid, 5% formaldehyde in water) overnight, washed in water and 70% ethanol and treated with 3% potassium hydroxide in water at room temperature for 1 week. After further dissection of the inner ear, the inner ears were cleared overnight in a mixture of glycerol: 70% ethanol: benzol (2:2:1) and photographed in 70% ethanol: benzol (1:1). Paintfilling of the inner ear was performed as described (Bissonnette and Fekete, 1996; Kiernan, 2006).

### In situ hybridisation and immunohistochemistry

Wildtype, *Cwe*/+, *Cwe/Cwe*, *Htu*+ and *Htu*/+; *Cwe*/+ embryos from timed matings were dissected in ice-cold PBS at E10.5 to E16.5, with E0.5 at noon the day the vaginal plug was found. For the marker analysis on sections 3 embryos of each genotype were used, for whole mount in situ hybrisation 4 embryos of each genotype were used. For whole mount in situ hybridisation embryos were fixed overnight at 4 °C in 4% paraformaldehyde in PBS and processed as described (Albrecht et al., 1997). For in situ hybridisation and immunohistochemistry on sections samples were fixed overnight at 4 °C in 10% neutral-buffered formalin, embedded in paraffin and cut into 8 μm sections and the Ventana Discovery system (Ventana Medical Systems, Inc Illkirch, France) was used according to the manufacturer's instructions. Plasmids containing cDNA of *Bmp4* (Jones et al., 1991), *Jag1* (Mitsiadis et al., 1997) and *Six1* (gift Dr. N. Bobola) and antibodies against Sox2 (Abcam, Cambridge, UK, cat. no. ab15830), Calretinin (Chemicon international, Millipore, Hampshire, UK, cat. no. AB5054), Myo7A (Proteus) and Jag1 (Santa Cruz, Heidelberg, Germany, cat. no. sc-6011) were used. The *NeuroD* in situ hybridisation probe was generated by RT-PCR on cDNA from wildtype E10.5 embryos (primer sequences: NeuroD-FW-T3 5′-AATTAACCCTCACTAAAGGGAGgttctcaggacgaggaacacgaggc-3′ and NeuroD-RV-T7 5′AATACGACTCACTATAGGGAGgcagcggcaccggaagagaagat-3′ followed by in vitro transcription using T7 polymerase to generate the antisense probe.

## Results

### Catweasel is a dominant mutation causing headbobbing due to a posterior crista defect

The catweasel (*Cwe*) mutation arose from a large-scale mutagenesis screen (Hrabé de Angelis et al., 2000). The catweasel founder was discovered because of its mild headshaking behaviour. Crossing the founder with wildtype C3HeB/FeJ mice showed that this phenotype had a dominant inheritance. *Cwe*/+ animals showed normal reaching, contact righting, and Preyer reflexes, normal swimming and spent normal time on the elevated platform and rotarod (data not shown). Whole skeletal preparations and analysis of the cleared adult inner ear and dissected middle ear revealed no obvious gross morphological differences between *Cwe*/+ mutants and controls (data not shown).

**Fig. 1:**
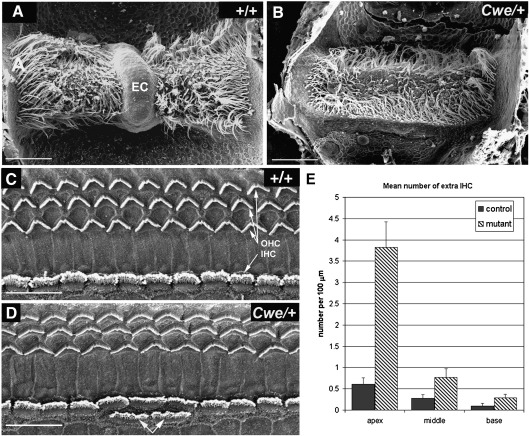
Catweasel mice have posterior crista defects and extra inner hair cells. (A, B) Scanning electron microscopy view of exposed posterior cristae of a wildtype (A) and a *Cwe*/+ (B) mouse. (C, D) Scanning electron microscopy view of the exposed organ of Corti of a wildtype (C) and *Cwe*/+ (D) mouse. (E) Quantification of the extra number of inner hair cells observed in wildtype (control) and *Cwe*/+ (mutant) mice per 100 μm of cochlear duct in the base, middle turn and apex. Standard errors are indicated. Scale bars: A–B = 50 μm; C–D = 25 μm. EC, eminentium cruciatum; IHC, inner hair cell; OHC, outer hair cell.

Scanning electron microscopy of the vestibular sensory epithelia showed no obvious abnormalities in the maculae, anterior and lateral cristae of *Cwe*/+ mice (data not shown). The posterior crista of wildtype mice normally has a non-sensory ridge running in the middle of the sensory patch, called the eminentium cruciatum (Desai et al., 2005; Fig. 1A). In the posterior cristae of *Cwe*/+ animals this eminentium cruciatum was missing (*n* = 11; Fig. 1B). There was no obvious difference in the overall size of the sensory region between *Cwe*/+ animals and controls, but the two ends of the sensory patch were more rounded in shape in *Cwe*/+ mice than in controls. Three animals scored as *Cwe*/+ by their headbobbing had an incomplete eminentium cruciatum, indicating that there might be a reduced penetrance for the posterior crista defect in *Cwe*/+ mice.

Scanning electron microscopic analysis of the organ of Corti showed that adult wildtype mice had a normal pattern of one row of inner hair cells (IHC) and 3 rows of outer hair cells (OHC). These hair cells all had well-developed stereocilia bundles (Fig. 1C). In *Cwe*/+ animals, hair cells developed normally and stereocilia were well-developed showing a normal staircase arrangement. However, we observed additional IHCs along the length of the cochlear duct (Fig. 1D). Counts of the ectopic second row of inner hair cells showed significantly more additional inner hair cells in *Cwe*/+ mutants than in wild type littermates in all turns of the cochlea, but especially in the apical turn (Fig. 1E, *Cwe*/+, *n* = 5 and +/+, *n* = 5).

### Catweasel mice have a point mutation in the *Six1* gene

**Fig. 2:**
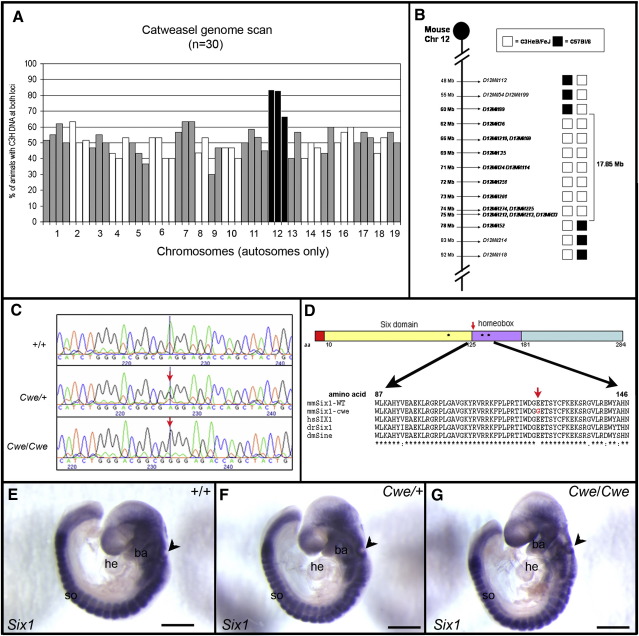
The catweasel mutation maps to chromosome 12 and catweasel mice have a mutation in the *Six1* gene. (A) Genome scan of 30 *Cwe*/+ animals. The percentage of animals with two C3H loci is displayed on the *Y* axis and alternating autosomes in white and grey on the *X*-axis with each bar representing one marker. (B) Schematical representation of the critical region on chromosome 12 between *D12Mit36* and *D12Mit274* (61.6–73.6 Mb). The two columns represent the genotypes of two mice with recombination breakpoints that defined the critical region (C) Partial sequencing of genomic DNA of wildtype, *Cwe*/+ and *Cwe/Cwe* mice using primers spanning exon 1 of the *Six1* gene. Between residue 220 and 230 of the sequenced PCR product, we identified an A to G substitution (red arrow) corresponding to position 411 of the *Six1* open reading frame. (D) A schematical representation of the Six1 protein, with its amino-terminal end (red), Six domain (yellow), DNA binding homeobox domain (purple) and putative transactivation domain (green). The amino-terminal region (between residues 87 and 146) of the homeobox domain is conserved between various species. The identified mutation in catweasel mice (red arrow) and residues mutated in branchio-oto-renal syndrome (⁎; Ruf et al., 2004) are indicated. (E–G) Wildtype (E), *Cwe*/+ (F) and *Cwe/Cwe* (G) embryos (E9.5) were analysed for *Six1* expression by whole mount RNA in situ hybridisation. *Six1* is expressed widely with high levels in somites and the otic cup (arrowhead). No significant difference between control and mutant embryos was found. Scale bar = 1 mm. aa, amino acid; ba, branchial arch; dmSine, *Drosophila melanogaster Sine oculis*; drSix1, *Danio rerio Six1*; hsSIX1, *Homo sapiens SIX1*; Mb, mega basepairs; mmSix1-Cwe, *Mus musculus Six1* with the catweasel mutation; mmSix1-WT, wildtype *Mus musculus Six1*; lb, limb bud; so, somites.

DNA from backcross offspring that exhibited severe headshaking behaviour was used to identify chromosome/trait linkage. Analysis of 57 polymorphic markers distributed throughout the autosomes indicated clear linkage of the catweasel behaviour to chromosome 12 (Fig. 2A). The highest percentage (83.3%) of homozygosity for the C3H-type polymorphism was found at marker *D12Mit69*. For fine mapping only backcross animals with a confirmed abnormal posterior crista were used (*n* = 38). This narrowed the interval to the region of chromosome 12 between *D12Mit36* and *D12Mit274*. The haplotypes of the two animals defining this critical region are shown in Fig. 2B. This corresponds to a 13 Mb physical region from 61.6 to 73.6 Mb on chromosome 12.

The region on chromosome 12 between *D12Mit36* and *D12Mit274* contains 82 genes (Ensembl Mouse V.39). Three members of the Six homeobox gene family, namely *Six1*, *Six4* and *Six6*, are clustered within this region. Both *Six1* and *Six4* are expressed in the developing inner ear from otic placode stages onwards. However only Six1 has been shown previously to be essential for dorsal-ventral patterning of the otocyst in mice and is involved in branchio-oto-renal (BOR) syndrome in humans (Ghanbari et al., 2001; Ruf et al., 2004; Zheng et al., 2003). We sequenced the exons of these *Six* genes, and did not find any mutations in *Six2* and *Six4* (data not shown). In exon 1 of the *Six1* gene we identified a substitution of A to G at position 411 (Fig. 2C).

The Six1 protein contains three domains, an amino (N-) terminal Six domain, involved in protein–protein interaction, a central Six type homeobox domain, essential for DNA binding, and at the carboxy (C-) terminus a putative transactivation domain. The identified mutation leads to an amino acid substitution from a glutamic acid (E) to a glycine (G) at residue 135 in the N-terminal part of the homeobox domain (Fig. 2D). This part of the homeobox is a loop that is identical between all mammalian Six proteins and highly conserved during evolution from *Drosophila melanogaster* to humans (Fig. 2D). As the catweasel mutation changes a negatively charged glutamic acid to a neutral glycine, it is likely that this causes destabilisation of DNA binding.

To assess the effect of the mutation on stability of the *Six1* mRNA, we analysed *Six1* mRNA expression at E9.5. In wildtype embryos *Six1* is widely expressed with high levels in somites and the developing inner (Fig. 2E; Laclef et al., 2003). We did not observe any differences in *Six1* expression between wildtype, *Cwe*/+ and *Cwe/Cwe* embryos.

Next we analysed the segregation of the heterozygous phenotype with the identified point mutation. From offspring of wildtype × *Cwe*/+ matings (*n* = 61), 42 animals (69%) were scored as *Cwe*/+ and 19 (31%) as wildtype on the basis of their headbobbing bahaviour. Genotyping by sequencing exon 1, showed that 29 animals (46%) were *Cwe*/+ and 32 animal were wildtype (53%). In two cases, a mouse was scored as wildtype by its behaviour but genotyped as *Cwe*/+. In these mice the posterior cristae were abnormal confirming the genotyping results. In 15 cases a mouse was scored as *Cwe*/+ by its behaviour, but sequencing clearly showed a wildtype genotype. For 11 of these mice we analysed the posterior crista, and these were normal. In the other 4 cases samples were lost or damaged during processing and we were unable to analyse the posterior crista. From these data we can conclude that the catweasel mutation segregates with the catweasel posterior crista phenotype. However, a mild headbobbing phenotype gives a relatively large number of false positives. Such behavioural observations must be confirmed with a morphological phenotype to allow mapping of mutations with weak behavioural effects.

### *Cwe/Cwe* animals are viable but have several defects

Next we analysed the offspring of *Cwe*/+ × *Cwe*/+ matings at postnatal days (P) 20–21. Out of a total of 88 animals, 21 animals (24%) were genotyped as wildtype and 47 (53%) as *Cwe*/+. An additional 20 animals (23%) displayed a more severe phenotype, exhibiting extreme headshaking and circling behaviour. Sequencing showed that these animals had a *Cwe/Cwe* genotype (data not shown). Wildtype and *Cwe*/+ mice had an average weight of 12–12.5 g (Table 1). *Cwe/Cwe* animals were small and significantly underweight (7.1 g +/− 1.4; *T*-test *P* = 1.79e^− 11^; Table 1). *Cwe/Cwe* animals performed abnormally in all behavioural tests performed. There was no reaching response when held by the tail, but instead the animals curled up towards their belly and displayed jiggling behaviour. There was neither a contact righting response nor Preyer reflex (data not shown). This indicated that the animals had inner and/or middle ear abnormalities resulting in vestibular dysfunction and hearing impairment.

**Fig. 3:**
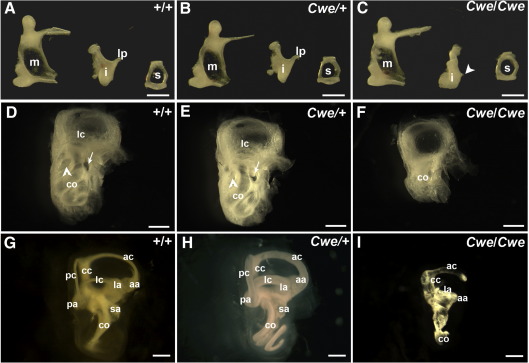
Mice homozygous for the catweasel have middle and inner ear defects. (A–C) Dissected middle ear ossicles from wildtype (A), *Cwe*/+ (B) and *Cwe/Cwe* (C) animals. The long process of the incus was absent in *Cwe/Cwe* mice (arrowhead). (D–F) Analysis of gross morphology of the inner ear of wildtype (A), *Cwe*/+ (B) and *Cwe/Cwe* (C) mice at P21 by glycerol clearing. (G–I) Analysis of gross morphology of the inner ear of wildtype (D), *Cwe*/+ (E) and *Cwe/Cwe* (F) embryos at E18.5 by paintfilling. Scale bars: A–C = 500 μm, D–F = 1 mm, G–I = 300 μm; m, malleus; i, incus; lp, long process incus; s, stapes; aa, anterior ampulla; ac, anterior semicircular canal; cc, common crus; co, cochlea; la, lateral ampulla; lc, lateral semicircular canal; pa, posterior ampulla; pc, posterior semicircular canal; sa, saccule.

We analysed these animals for gross malformations and found that 41% of *Cwe/Cwe* animals had a unilateral hypoplastic kidney (Table 1; data not shown). Analysis of the middle ear showed that wildtype (*n* = 2) and *Cwe*/+ (*n* = 4) animals had a normal malleus, incus and stapes (Figs. 3A, B). *Cwe/Cwe* animals (*n* = 4) had a normal malleus and stapes, but lacked the long process of the incus (Fig. 3C). Analysis of sections through embryos showed that the long process is not present in *Cwe/Cwe* embryos at E16.5 (data not shown).

### *Cwe/Cwe* animals have canal abnormalities and a truncated cochlea

The gross morphology of the inner ear at P21 was assessed by glycerol clearing. Wildtype and *Cwe*/+ animals have normal morphology (Figs. 3D, E). Inner ears of *Cwe/Cwe* animals were smaller than controls (Fig. 3F). There was a clear vestibular part visible and a structure similar to the base of the cochlea appeared to be present. Although a rudimentary vestibular system was present, the lateral semicircular canal was absent, and other canals were thin or truncated. The cochlea was severely truncated and oval and round windows were absent.

To analyse the inner ear malformation in more detail we analysed paint filled inner ears of E18.5 embryos. Wildtype and *Cwe*/+ animals at this stage had a normal morphology of the inner ear (Figs. 3G, H). The inner ear of *Cwe/Cwe* animals was smaller, but vestibular and cochlear parts were present (Fig. 3I). The lateral and posterior semicircular canals were absent. The anterior canal was present but truncated at the ampullar end. The anterior and lateral cristae ampularis were present, but the posterior crista was absent. The common crus was broadened and the cochlea was severely truncated and malformed. The utricle and saccule were smaller in size than in control animals.

### *Cwe/Cwe* animals lack most hair cells

**Fig. 4:**
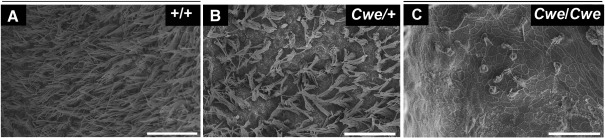
Analysis of the sensory epithelium of the utricle by scanning electron miscroscopy. (A–C) Scanning electron microscopy view of the exposed sensory epithelium of the utricle of wildtype (A), *Cwe*/+ (B) and *Cwe/Cwe* (C) animals. Scale bar: 30 μm.

**Fig. 5:**
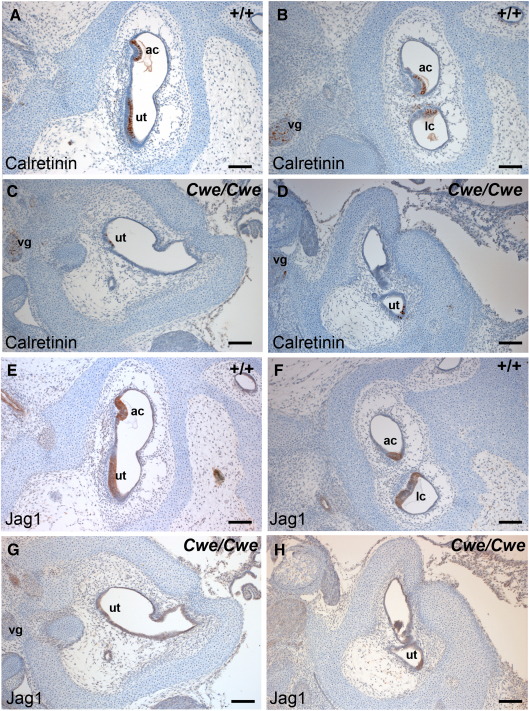
Immunolabelling of adjacent sections for Calretinin (A–D) and Jag1 (E–H) shows that *Cwe/Cwe* animals lack most hair cells in the inner ear. (A, B) Calretinin-positive hair cells have developed in the utricle and cristae of wildtype animals at E16.5. In addition, calretinin marks the nuclei in the vestibular ganglion (C–D) Most sections through the *Cwe/Cwe* inner ear were devoid of hair cells but in a few sections very few calretinin-positive cells are found. The vestibular ganglion marked by calretinin expression appears normal. (E, F) At E16.5 wildtype inner ears have Jag1-positive sensory patches. (G, H) *Cwe/Cwe* animals have decreased Jag1 labelling. Scale bar = 200 μm. ac, anterior crista; lc, lateral crista; ut, utricle; vg, vestibular ganglion.

As the anterior and lateral cristae ampulari and a rudimentary base of the cochlea were present in *Cwe/Cwe* animals, these structures were analysed by scanning electron microscopy at P21. Examination of the cristae ampulari and rudimentary cochlea revealed that no sensory hair cells were present in *Cwe/Cwe* animals (data not shown). However the utricle contained a small patch of scattered hair cells (Figs. 4A–C). To determine whether this deficit of hair cells is due to a defect in hair cell development or due to hair cell degeneration, we analysed serial sections of the inner ear of E16.5 embryos stained for Calretinin. Calretinin normally marks the hair cells in cristae, maculae and organ of Corti (Dechesne et al., 1993; Figs. 5A, B; data not shown). *Cwe/Cwe* embryos had very few scattered calretinin-positive cells in two small patches (Figs. 5C, D). Due to the abnormal gross morphology of the *Cwe/Cwe* inner ear it is difficult to establish in which areas these cells are located. Based on our scanning electron microscopical observations at P21 we propose that it is likely that these cells are located in the utricle. The Notch ligand *Jag1* is expressed throughout the sensory patch from E10.5 onwards, and is essential for sensory progenitor development in the mammalian inner ear. At later stages of development Jag1 expression becomes restricted to the supporting cells (Lewis et al., 1998; Kiernan et al., 2001, 2006). At E16.5 Jag1 protein is localised throughout the sensory epithelium, including immature hair cells in wildtype embryos (Figs. 5E, F). In the *Cwe/Cwe* inner ear at this stage, we observed a very faint Jag1 staining in the area that also contained the few Calretinin positive cells (Figs. 5G, H). This indicates that the great reduction in hair cells at P21 is due to a failure in hair cell development rather than hair cell loss.

### *Six1* acts early in the prosensory pathway

**Fig. 6:**
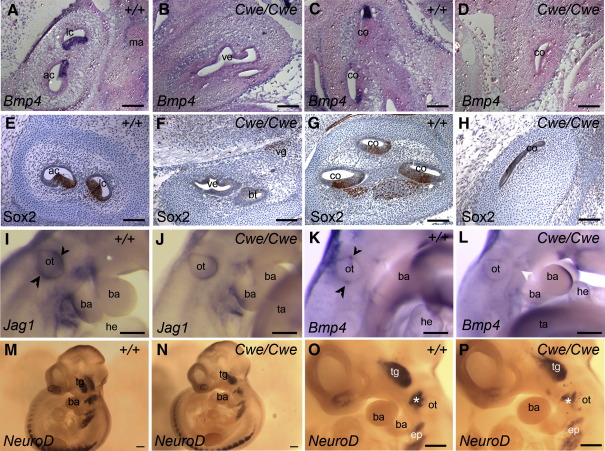
Sensory patch and cranial neurogenic development is affected in *Cwe/Cwe* mice. (A–D) RNA in situ hybridisation for *Bmp4* on E14.5 sections. (A) *Bmp4* marks the developing cristae in control mice. (B) No *Bmp4* expression is detected in *Cwe/Cwe* vestibular system. (C) In the wildtype cochlea, *Bmp4* is expressed adjacent to the developing organ of Corti. (D) No *Bmp4* expression was detected in *Cwe/Cwe* cochlea. (E–H) Immunolabelling for Sox2 on E14.5 sections. (E) In wildtype embryos Sox2 marks the all sensory patches including the cristae shown here and the vestibular ganglion (not shown). (F) *Cwe/Cwe* animals have normal Sox2 expression in the vestibular ganglion, but lack Sox2 in the vestibular epithelia. (G) In wildtype embryos Sox2 marks the developing organ of Corti and the acoustic ganglion. (H) No Sox2 positive cells were found in the severely truncated *Cwe/Cwe* cochlea. (I–L) Whole mount RNA in situ hybridisation for *Jag1* and *Bmp4* at E10.5. (I) In wildtype embryos, *Jag1* was detected in an anterior and posterior expression domain in the otocyst (arrowheads). (J) *Cwe/Cwe* embryos lack *Jag1* expression in the anterior and posterior patches of the otocyst. In contrast, a ventral expression was detected. (K) *Bmp4* expression marks anterior and posterior patches in the wildtype otocyst (arrowheads). (L) In *Cwe/Cwe* embryos *Bmp4* expression is absent in the otocyst. (M–P) Whole mount RNA in situ hybridisation for *NeuroD* at E10.5. (M, O) *NeuroD* expression marks all cranial neurogenic placodes including the olfactory placode (delineated), epibranchial and the vestibulo-acoustic placode (⁎). (N, P) Stage-matched *Cwe/Cwe* embryos have significant smaller *NeuroD*-expressing olfactory (delineated), epibranchial and vestibulo-acoustic (⁎) domains. Scale bar = 200 μm. ac, anterior crista; ba, branchial arch; bt, basal turn of cochlea; co, cochlea; ep, epibranchial; he, heart; lc, lateral cristal; ma, malleus; ot, otocyst; ta, tail; tg, trigeminal ganglion; ve, vestibule; vg, vestibular ganglion.

To establish the role of *Six1* in the development of the sensory patch, we analysed the expression of several markers for the sensory patch at earlier stages. The growth factor *Bmp4* is expressed in the developing cristae and in the cochlea at E14.5 (Figs. 6A, C; Morsli et al., 1998). In addition, *Bmp4* is expressed in various other tissues, including the condensing mesenchyme around the inner ear and around the developing ossicles (Figs. 6A,C). *Cwe/Cwe* animals did not have any *Bmp4*-expressing cells in the vestibule and cochlea (Figs. 6B, D). However, *Bmp4* expression was normal in other tissues including the mesenchyme around the inner ear (Fig. 6B; data not shown).

The transcription factor Sox2 is a marker for the sensory patch and has been shown previously to be essential for sensory patch development (Kiernan et al., 2005). At E14.5 Sox2 marks all the sensory patches and the developing neurons that will be innervating the sensory hair cells (Figs. 6E, G). *Cwe/Cwe* embryos have no Sox2 positive areas in the vestibular and cochlear parts of the inner ear at this stage (Figs. 6F, H). Furthermore, we did not detect any Sox2 positive neurons in the developing inner ear, suggesting that these are absent (Figs. 6F, H).

At E10.5 *Jag1* expression marks an anterior and posterior area of the otocyst that is thought to give rise to the sensory patches (Fig. 6I; Daudet and Lewis, 2005; Kiernan et al., 2006). In addition *Jag1* is expressed in branchial arches, limb buds and the developing nervous system (Fig. 6I; data not shown). A clear dorso-ventral axis in the otocyst is present at this stage in both wildtype and *Cwe/Cwe* embryos (Figs. 6I, J). However at E10.5 *Cwe/Cwe* animals can be distinguished from their wildtype and *Cwe*/+ littermates by a slightly enlarged and less pointed endolymphatic compartment. *Cwe/Cwe* embryos have *Jag1* expression in branchial arches, limb bud and nervous system (Fig. 6J; data not shown), but only a faint, ventral *Jag1* expression domain in the developing inner ear (Figs. 6I, J). We did not detect any *Jag1* in anterior or posterior patches of the *Cwe/Cwe* inner ear. Similar to *Jag1*, *Bmp4* marks the anterior and posterior patches in the otocyst at E10.5 (Fig. 6K; Gerlach-Bank et al., 2004). In addition it is also expressed in branchial arches, limb bud and other tissues (Fig. 6K; data not shown). *Cwe*/+ animals have a normal expression of *Bmp4* in the otocysts and other tissues (data not shown). *Cwe/Cwe* animals have normal expression in branchial arches and limb bud, but no expression was detected in the otocyst (Fig. 6L). The lack of *Jag1* and *Bmp4* expression in the anterior and posterior prosensory patches at E10.5 indicates that the development of the sensory patches is affected at very early stages in *Cwe/Cwe* animals.

*Six1* has been shown to be essential for neurogenic development in the olfactory placode (Ikeda et al., 2007; Chen et al., 2009) but has been described to inhibit the formation of zebrafish otic neurons (Bricaud and Collazo, 2006). As *Cwe/Cwe* embryos have a loss of Sox2-positive neurons in the developing inner ear at E14.5 we analysed the development of neurogenic placodes at E10.5 (Figs. 6M–P). *NeuroD* marks all cranial and epibranchial placodes in wildtype embryos (Figs. 6M, O). In stage-matched *Cwe/Cwe* embryos all placodes are present, but there is a reduction in size especially of the olfactory, vestibulo-acoustic and epibranchial placodes (Figs. 6N, P).

### *Six1* interact genetically with the Notch ligand *Jag1*

The eminentium cruciatum defect and extra inner hair cells observed in *Cwe*/+ mice are very reminiscent of defects seen in *Jag1* heterozygous mutant (headturner, *Htu*/+) mice (Kiernan et al., 2001). To test for genetic interaction we bred *Cwe*/+ and *Htu*/+ animals. *Htu*/+ animals appear to have reduced fitness, but we did recover normal Mendelian ratios of offspring from crosses between *Htu*/+ and *Cwe*/+ animals (Table 2). All seven double adult heterozygote animals (*Htu*/+; *Cwe*/+) had headbobbing behaviour that is typical for *Htu*/+ mice. However *Htu*/+; *Cwe*/+ mice displayed circling behaviour, a feature never observed in single *Cwe*/+ or *Htu*/+ mutant mice (Suppl. Data Mov. 1,2,3).

**Fig. 7:**
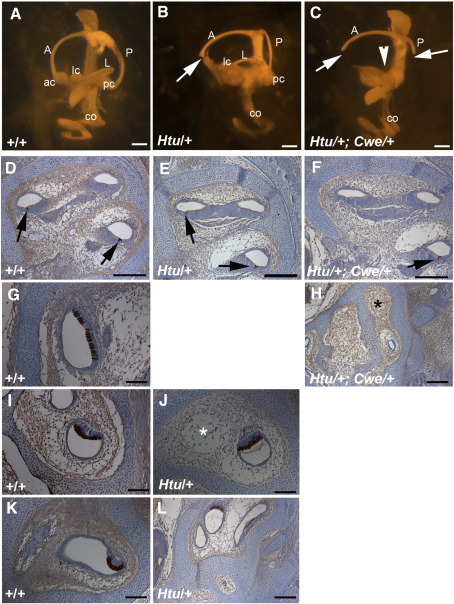
Mice double heterozygous for the catweasel and headturner mutation have truncations of all three semicircular canals and lack the cristae. (A–C) Analysis of gross morphology of the inner ear of wildtype, *Htu*/+ and *Htu*/+;*Cwe*/+ mice at E16.5 by paintfilling. (A) Wildtype embryos have a fully developed inner ear with clearly distinguishable semicircular canals and cristae. (B) *Htu*/+ embryos always have anterior canal truncations (white arrow) and sometimes posterior canal truncations (not shown). (C) *Htu*/+;*Cwe*/+ embryos always have anterior and posterior canal truncations, and in addition have lateral canal defects (arrowhead pointing to area where the lateral semicircular canal is missing). No cristae ampulare can be identified (D–L) Sections through the inner ear stained for Myo7a protein expression of wildtype, *Htu*/+ and *Htu*/+;*Cwe*/+ embryos at E16.5. (D–F) Sections through the cochlea of a wildtype, *Htu*/+ and *Htu*/+;*Cwe*/+ embryos shows normal gross morphology. (G) A section through a wildtype anterior crista expressing Myo7a. (H) A section through the vestibular system of a *Htu*/+;*Cwe*/+ embryo. No Myo7a-positive cristae could be identified. Note the absence of the anterior canal (⁎). (I) A Myo7a-positive lateral crista in a wildtype embryo. Note on the top left the anterior canal is visible (J) *Htu*/+ embryos have normal lateral cristae. Note the absence of the anterior canal (⁎). (K) A section through an wildtype posterior crista expressing Myo7a. (L) This *Htu*/+ embryo lacks the posterior crista. Scale bars: A–C = 1 mm, D–L = 200 μm. A, anterior semicircular canal; ac, anterior crista; co, cochlea; L, lateral semicircular canal; lc, lateral crista; P, posterior semicircular canal; pc, posterior cristae.

We analysed the anatomy of adult inner ears by inner ear clearing (data not shown) and of embryos by paintfilling (Figs. 7A–C). Confirming our earlier observations, wildtype and *Cwe*/+ animals showed no obvious gross inner ear abnormalities and *Htu*/+ animals had truncations of anterior and posterior canals but normal lateral canals (data not shown; Figs. 7A,B) (Kiernan et al., 2001). *Htu*/+; *Cwe*/+ mice and embryos had apparently normal cochleas but had truncations of the anterior and posterior semicircular canals (Fig. 7C and data not shown)). In addition all double heterozygous mice had lateral canal truncations in both ears and no obvious cristae ampulare could be detected (Figs. 7C, F and data not shown).

In addition we analysed serial sections from E16.5 embryos stained for Myo7a (Figs. 7D–L). Myo7A protein expression marks the developing hair cells in the cochlea, maculae and cristae in wildtype embryos (Figs. 7A, D, G, I, K). We did not detect any obvious gross malformation of the cochlea of *Htu*/+; *Cwe*/+ embryos compared to wildtype and *Htu*/+ embryos (Figs. 7D–F). *Htu*/+ embryos were always lacking the anterior cristae and sometimes the posterior cristae (Figs. 7J, L). In *Htu*/+; *Cwe*/+ inner ears, we failed to identify any Myo7A-positive cristae (Fig. 7H).

This synergy between *Six1* and *Jag1* mutations in producing a lateral canal truncation and circling behaviour, suggests that these two genes act in the same pathway during inner ear development.

## Discussion

### Catweasel is a novel hypomorphic mutation in *Six1* gene

Catweasel is a novel ENU-induced mutation causing a mild headbobbing in heterozygote mice. Here we showed that this is associated with a very mild posterior crista defect, namely an abnormal shape of the crista and the absence of the eminentium cruciatum. We propose that a mutation in the *Six1* gene is likely to be the causative mutation in catweasel mice as 1) the *Six1* gene lies in the catweasel critical region, 2) the identified mutation segregates with the catweasel phenotype, 3) the identified mutation changes a highly conserved, charged residue into a neutral residue, 4) all tissues affected in catweasel mice express *Six1* during embryogenesis, 5) the catweasel phenotype is similar, though not identical, to defects described in *Six1* null mice and 6) the phenotype of catweasel mice is very similar to defects found in BOR syndrome, which can be caused by mutations in *SIX1*.

We have mapped the catweasel mutation to a region on chromosome 12 between *D12Mit36* and *D12Mit274*. Of the 89 genes in this region, three Six genes (*Six1*, *Six2* and *Six4*) were the most likely candidate genes based on gene expression pattern and previously reported phenotype (Zheng et al., 2003; Ozaki et al., 2004). By exon re-sequencing we identified a point mutation (A411G) in the *Six1* gene. The identified mutation leads to an amino acid substitution from a glutamic acid (E) to a glycine (G) at residue 135 in this conserved amino terminal part of the homeobox domain. It has been shown that single amino acids can determine the specificity of DNA binding of homeodomain proteins (Treisman et al., 1989). The catweasel mutation changes a negatively charged glutamic acid to a neutral glycine. Most *SIX1* mutations identified in humans are located close to the residue mutated in catweasel mice (⁎ and arrow in Fig. 2D; Ito et al., 2006; Ruf et al., 2004). Mutations found include a point mutation changing one amino acid in the Six domain (T110W) and two types of point mutations affecting a single residue in the amino-terminal loop of the homeodomain (Y129C and delE133) (Ruf et al., 2004; Ito et al., 2006). Ruf and colleagues have shown that the mutations identified in the homeodomain affect binding of SIX1 to the MEF3 site. Whereas the Y129C mutation only reduces DNA binding, the delE133 mutation completely abolishes binding of SIX1 to the MEF3 site. In addition, all three mutations also affected interaction with EYA1. To date we have not tested the effect of the catweasel mutation on DNA binding and Eya1 interaction, but based on the type of substitution and the similarities to the mutations reported by Ruf et al (Ruf et al., 2004) it is likely that both DNA binding and Eya1 interaction are affected.

The crucial role for mouse *Six1* in the development of the inner ear, kidney and other organs has been described previously (Laclef et al., 2003; Xu et al., 2003; Zheng et al., 2003; Ozaki et al., 2004). In contrast to the mutation described in this study, these groups used targeted mutations, which are likely null mutations. Observations made by these groups strongly suggest that *Six1* has dose-dependent effects on the development of the inner ear and kidney. Complete loss of *Six1* leads to perinatal lethality due to abnormalities in several organs (Laclef et al., 2003; Xu et al., 2003). In these mice inner ear development arrests at the otocyst stage associated with a dorso-ventral patterning defect and there is an absence of the kidneys at birth. Mice heterozygous for the null mutation have milder inner ear malformations. These include absence or malformation of the endolymphatic sac, truncation of the cochlea and mild saccule abnormalities (Zheng et al., 2003). In addition, some heterozygote mice have kidney defects that are genetic background-dependent (Xu et al., 2003). Reducing the dose of *Six1* gives rise to a spectrum of inner ear and kidney defects. The spectrum of the phenotype is as follows (from normal to very severe defects): *Six1*^*WT*^ < *Six1*^*Cwe*^^/+^ < *Six1*^*+/null*^ < *Six1*^*Cwe/Cwe*^ < *Six1*^*null/null*^ (Laclef et al., 2003; Xu et al., 2003; Zheng et al., 2003; Ozaki et al., 2004) (and this study).

Catweasel heterozygote mice have a distinct inner ear phenotype that is less severe than the phenotype described in *Six1*^+/null^ mice. In *Cwe*/+ mice the kidneys do not appear to be affected but we have not investigated genetic background effects. In contrast, the phenotype of catweasel homozygote mice appears more severe than the phenotype in *Six1*^+/null^ mice, but milder than the phenotype in *Six1* null mice. In catweasel homozygote mice, we observed the formation of the vestibule and a (strongly truncated) cochlea indicating that dorso-ventral patterning is normal. The kidney defect of catweasel mice is also less severe than the null, but more severe than the *Six1*^+/null^ state. From this we conclude that the catweasel mutation is likely a hypomorphic mutation.

### *Six1* plays a crucial role in inner ear morphogenesis and sensory patch specification

*Six1* expression in several model organisms suggest that *Six1* could act at several crucial stages of inner ear development from specification of the otic placode to hair cell development (Zheng et al., 2003; Bessarab et al., 2004; Schlosser and Ahrens, 2004). *Six1* has been shown to play a crucial role in the D-V patterning of the otocysts in mouse (Xu et al., 2003). At E10.5 *Six1*^null/null^ inner ears do not show any sign of endolymphatic duct formation. At later stages of development, a rudimentary endolymphatic sac and duct are present, but all other structures including cochlea and semicircular canals fail to form. These observations combined with marker analysis have indicated that *Six1* plays a crucial role in establishing the ventral part of the otocyst, which is consistent with the ventral expression of *Six1* at E9.5. In contrast to the null phenotype, the inner ear of catweasel homozygous animals can be distinguished from their wildtype and *Cwe*/+ littermates by a slightly enlarged and less pointed endolymphatic compartment. At later stages, several vestibular structures (anterior semicircular canal, common crus, anterior and lateral ampullae) and a rudimentary cochlea are present.

Defects observed in *Cwe/Cwe* mice include absence or truncation of semicircular canals, broadened common crus and truncated cochlea. These defects are linked to the very specific expression pattern of *Six1* in the developing sensory epithelia of the vestibular system and cochlea from mid-gestation onwards (Zheng et al., 2003). Although some ampullae are present in *Cwe/Cwe* mice, these are devoid of any hair cells. The only sensory epithelium that contained any hair cells was the utricle. Absent or abnormal expression of early markers for sensory patch development, including Sox2, *Bmp4* and *Jag1*, suggests that *Six1* is upstream of these genes in the prosensory cascade, consistent with observations made in zebrafish (Bricaud and Collazo, 2006).

Truncations of the semicircular canals are often linked to cristae defects, implying that interaction between these tissues is crucial for normal development of the vestibular system (Whitfield et al., 1996; Anagnostopoulos, 2002; Burton et al., 2004; Chang et al., 2004; Kiernan et al., 2005). Experiments in the chicken have led to the model where FGFs secreted from the sensory patch are crucial for growth and development of the semicircular canals (Chang et al., 2004). Therefore, the canal truncations observed in catweasel homozygous mice are likely to be secondary due to absence of the sensory patch.

### The role of *Six1* in cranial placode-derived neurogenic differentiation

Several publications have addressed expression and function of *Six1* during placodal development. *Six1* expression marks all cranial placodes in *Xenopus laevis* and zebrafish and has been proposed to promote placodal fate (Ghanbari et al., 2001; Bessarab et al., 2004; Brugmann et al., 2004; Schlosser and Ahrens, 2004). Other studies showed that *Six1* is essential for neurogenic development in the olfactory placode (Ikeda et al., 2007; Chen et al., 2009). In addition, vestibulo-acoustic neuroblasts express *Six1* from when they delaminate from the otocyst until at least E12.5 and the vestibule-acoustic ganglion is absent in *Six1*^null/null^ embryos (Zheng et al., 2003). These data suggest that *Six1* has a pro-neurogenic function. In contrast, Bricaud and Collazo (2006) postulated that *Six1* promotes mechano-sensory hair cell fate at the expense of neurogenic fate in zebrafish. These apparently contrasting roles have been dissected out in more detail in a recent study on the role of *Six1* and *Eya1* in placodal development (Schlosser et al., 2008). Schlosser et al. found that *Eya1* and *Six1* are crucial to promote neurogenesis at early stages. During neurogenic differentiation, high levels of *Six1* maintain neurogenic progenitors in a proliferating undifferentiated state, whereas lower levels of *Six1* allow for exit of the cell cycle and neurogenic differentiation to occur. Here we showed that *Cwe/Cwe* mice have a reduction in vestibulo-acoustic neuroblasts at E10.5, confirming the pro-neurogenic role of *Six1*. In contrast to the *Six1*^null/null^ mutation, some neuroblasts are forming in *Cwe/Cwe* animals, confirming again the hypomorphic nature of the mutation.

### Six1 may act upstream of Notch signalling in various stages of sensory patch development

*Six1* potentially may have a role in the later steps of sensory patch differentiation. Catweasel heterozygous animals have very specific defects that include the absence of the eminentium cruciatum of the posterior crista and the extra inner hair cells throughout the organ of Corti. This is very reminiscent of defects seen in *Jag1* heterozygous mutant mice (Kiernan et al., 2001), which have extra inner hair cells and absence of the posterior crista combined with either absence of the eminentium cruciatum in the anterior crista or complete absence of the anterior crista. The similarity in the phenotype between catweasel and headturner mice suggests that there could be an important link between Six1 and Notch-Jag1 signalling in the differentiation of hair and supporting cells in the sensory patch by lateral inhibition. The genetic interaction of *Six1* and *Jag1* provides further evidence for this.

We showed that *Jag1* expression in the early sensory patches is absent in *Cwe/Cwe* embryos, suggesting that *Six1* is upstream of *Jag1* in sensory patch development. Analysis of conservation of non-coding DNA around the *Jag1* locus has identified conserved areas that could acts as enhancer elements (http://enhancer.lbl.gov/cgi-bin/enh.pl?keyword=jag1&form=searchGene&action=search). These elements contain many transcription factor (including homeobox) binding sites. Six1 binding to several known promoters have been studied by various groups, but no unique binding sequence has been identified (Ruf et al., 2004; Ando et al., 2005). Therefore future work is needed to identify Six1 binding sites in the *Jag1* promotor.

### *Cwe/Cwe* mice are a good model for branchio-oto-renal syndrome

BOR syndrome caused by *EYA1*, *SIX1* and *SIX5* mutations leads to kidney and urinary tract malformations and hearing impairment (Melnick et al., 1976; Abdelhak et al., 1997; Ruf et al., 2004; Hoskins et al., 2007). Detailed radiological analysis of the inner ear malformations in BOR syndrome is scarce. Ceruti et al. used computed tomography and MRI on temporal bones of eight affected members from one family with BOR syndrome (Ceruti et al., 2002). Cochlear malformations were identified in all patients. Abnormalities included dysplasia or hypoplasia of the cochlea. Ceruti et al. described in detail vestibular abnormalities that occur in seven of these patients. These included enlarged endolymphatic sac and duct and lateral and/or posterior canal truncations (Ceruti et al., 2002). To date, no anterior (superior) semicircular canal abnormalities have been reported in BOR syndrome. Mice homozygous for the *Six1*^*Cwe*^ mutation have severe truncation of the cochlea, combined with canal malformations and enlarged endolymphatic sac. The lateral and posterior canals are severely affected (absent) whereas the anterior canal is present but trunctated anteriorly. These defects are highly similar but slightly more severe than those described in BOR syndrome.

Middle ear defects described in BOR syndrome include hypodysplastic stapes, malleus–incus fusion and closed oval windows (Ceruti et al., 2002). This middle ear phenotype is very variable between family members and even between the left and right ear. Catweasel homozygote mice lacked the long process of the incus in both ears, likely causing a disruption of the ossicular chain. In addition we found absence of the oval and round windows in most animals. The absence of other middle ear abnormalities could be due to the homogeneous (inbred) genetic background of the mice. In humans bilateral congenital absence of the long process of the incus is rare. Necrosis of the long process later in life can be due to infections, trauma and diabetes mellitus (Tüz et al., 2006). The bilateral absence of the long process in catweasel homozygote mice at embryonic stages suggests that this is a developmental defect, and not due to necrosis of the long process after birth.

BOR syndrome is characterised by various renal abnormalities leading to end-stage renal disease at variable age due to unilateral renal agenesis with contralateral or bilateral hypoplasia (Pierides et al., 2002). A large proportion of catweasel homozygote mice display unilateral kidney hypoplasia at postnatal day 21. The few animals that were kept until postnatal day 28 did show abnormal coloration of the kidneys, suggesting that these mice develop a type of end-stage renal disease (data not shown).

Patients with BOR syndrome are heterozygous for the causative mutation. Whereas *Cwe*/+ mice are relatively normal, the *Cwe/Cwe* phenotype closely resembles the phenotype observed in BOR syndrome. The catweasel mutation might have a less severe affect on DNA binding and/or Eya1 interaction than BOR mutations described to date or genetic background effects might account for the difference in heterozygous phenotype. The phenotype of the *Six1* null mouse is more severe than the phenotype in BOR patients and targeted *Six1* heterozygote mice have a phenotype less severe than BOR syndrome. As we found a strong overlap in phenotype between BOR syndrome and *Cwe/Cwe* we propose that these mice provide a useful model to study the mechanisms underlying defects in BOR syndrome in more detail.

## Figures and Tables

**Table 1:** Summary of the analysis of the catweasel homozygous phenotype at P21

	Weight				Phenotype		
	*n*	Average (g)	StDev	Truncated cochlea (%)	Affected SSC (%)	Kidney abnormalities (%)	Affected incus (%)
+/+	15	12.0	0.6	0	0	0	0
*Cwe*/+	19	12.5	1.5	0	0	0	0
*C/C*	12	7.1⁎	1.4	100	100	41	100

g, grams; *C/C*, *Cwe/Cwe*; SSC, semicircular canal; StDev, standard deviation; WT, wildtype.

⁎ T-test statistically significant with P*T*-test statistically significant with *P* < 0.001.

**Table 2:** Summary of test for Mendelian ratios in catweasel, headturner and intercrosses

Genotype offspring							Chi square
Mating	*n*	+/+;+/+	*H*/+;+/+	+/+;*C*/+	*H*/+;+/*C*	+/+;*C/C*	*P* < 0.05
	183	89		94			No
*C*/+ × *C*/+	88	21		47		20	No
*H*/+ × +/+	193	128	76				Yes
*H*/+ × *C*/+	45	13	13	12	7		No

*C*/+, *Cwe*/+; *H*/+, *Htu*/+.
