# Supplementary material for: Catweasel mice: A novel role for Six1 in sensory patch development and a model for branchio-oto-renal syndrome
Source: Dev Biol. 2009 Apr 15;328(2):285–96. doi: 10.1016/j.ydbio.2009.01.030 (PMC2682643; doi:10.1016/j.ydbio.2009.01.030)
Supplement: Supplementary Materials [file mmc4.doc]

**Supplementary table I**

| **Locus** | **cM** | **FW** | **RV** |
| --- | --- | --- | --- |
| D1Mit21 | 33.89 | CGCTGGACAATCTTATAATTGCA | TCGAATCCCAACAACCACAT |
| D1Mit415 | 53.06 | TTGGCACATGCCTACAACTC | AGAACACCATATATTGTGCCCC |
| D1Mit445 | 75.72 | GGAGATTGCATGTCCTTTTAGG | GTACACAGGCGCACACATTC |
| D1Mit353 | 90.34 | TACACTATGGGTATATGCTCACTATGC | ACACATGAACATACTCATATGCACA |
| D2Mit237 | 28.71 | TTCCAAGTCACCTATTATCAAAAGG | TTGATACGGACACCAGCAAA |
| D2Mit128 | 49.41 | ACCATGAGGGCAGAATTCAC | CTAGAGGGTTTGCTGCCTTG |
| D2Mit200 | 98.36 | ATGGCCTCTGCTAAATGGTG | GCTAGCAGGAGCGTCATAGG |
| D3Mit117 | 2.35 | TTCTGGAGGCAAGATTGAGG | TCATTTTTCTATTCTGTTTAGTGTGTG |
| D3Mit339 | 29.25 | TCTATATTTGGGGGGAAGGG | GATTTAGTGTCAAAGGCTATGCA |
| D3Mit199 | 56.1 | CCAGACCTCAGAAAGTGAGTCC | ACCATGACATTCATGCTATTGTG |
| D4Mit172 | 11.23 | TGCAGGTAGGGGCTCAAG | AAGCAGATCTGGCTTCCTCA |
| D4Mit58 | 40.62 | TATTTTTTGGGTTTGGAAGGG | CCTTGCAGCCACACTCAGT |
| D4Mit33 | 67.6 | GGAGGTGTCAGGAGCCCT | CTCCTGACTGAGGTACCAAACC |
| D5Mit345 | 0 | ATCTTGATCCTCCTGCTTTAACC | CAAAGTTGCCACAGATATTTGC |
| D5Mit391 | 18.03 | AATAAGAAAATTCCACCAAGTCTACA | CTTGATGGGTCTGATGCCTT |
| D5Mit115 | 40.01 | AGAAAAGCTGTTGCCTGCTC | CAAAGCTGAAAGAAACAAAGATATC |
| D5Mit168 | 68.65 | CAGGTGACAGTTGTTCTCTTCC | CATGCATGAACACACATCACA |
| D6Mit138 | 2.35 | GCTCTTATTAATGAAGAAGAAGGAGG | CAAAGAAAGCATTTCAAGACTGC |
| D6Mit320 | 22.38 | TCTTAAAGATAGTATCATGCATGTGTG | CTGGAGACAGAACTATTTCCTTCC |
| D6Mit366 | 43.69 | AAGGCTCTGGTTTGCTAATAACC | GACCTTTTTACAAAGTTTAGGTCCC |
| D6Mit201 | 62.94 | TGCTTCCTCTCTGCTGTAAGC | AACTAAGGCCAGTACTGAAAAGTACA |
| D7Mit178 | 2.78 | ACCTCTGATTTCAGAACCCTTG | TAGAGAGCCACTAGCATATCATAACC |
| D7Mit230 | 22.45 | GGGTTAACTGCTTTTTAAAAGTGC | ACTTCTGCATGTTGCCCTCT |
| D7Mit253 | 42.55 | TGTGGGTGCAACCAAATG | TTTGGTGATATAGATACTAGGTGTGTG |
| D8Mit190 | 22.69 | CTTTGTTGCTGTTTCATTCTGG | AGTCATATACAAGGTCAACCTGAGC |
| D8Mit280 | 74.11 | CATGCAATTCCAATGTCAGTG | TAGCACTCAATCAAACCCCC |
| D9Mit254 | 20.14 | TTCTCCTTTCTTTTCACTAGTGTGC | TGATGCCTCTGGTATCCACA |
| D9Mit214 | 58.79 | AGCACAGGAAAAGGACGCTA | AACCTGTCTCTGTAAAACTATCTCCA |
| D10Mit206 | 4.47 | AAGACCCATTCATATCCCTAGTT | TGCTAACCAAGAAAAGAGAGTCG |
| D10Mit115 | 32.47 | CCATGGAATAGAAGTCTTTAAGAAGC | ACCTGAAGGATAAAGGGTCTTACC |
| D10Mit180 | 65.22 | GACCTTCCTTTATACACAAGTCATAGC | GTGGTACAGAACTTAGGTGTTTAATTG |
| D11Mit71 | 0 | GCCATACCTGGTAGCGTGTT | AATTTTCAGATGTAGCCATAAGCC |
| D11Mit140 | 25.73 | GCATTTACTTGATTGATTGTTTGC | ACCCAATGCCTGCCTCTAC |
| D11Mit35 | 44.74 | AGTAACATGGAACATCGACGG | TGCTCAGCTCTGGAGTGCTA |
| D11Mit99 | 63.21 | CTGTAGGTAAAATACACTTGCCG | GGTGGACAGACCCTTCTGAA |
| D11Mit214 | 79.6 | CATACAGCCTTCAACAATGACA | ACTGCATACATGTGCACTCATG |
| D12Mit69 | 23.88 | GAAGAGAGGACATTGCACTGG | AGTTACTGAAGCATAGACCAACCC |
| D12Mit259 | 42.92 | TACCTTGAGAAAAGTATGGAGAAATG | TAGCAACATGTAAAAGCATGATACC |
| D12Nds2 | 62.46 | ACATGGTAATTTATGGGCAA | CTGGATACCTGCAATAGTAGA |
| D13Mit3 | 8.96 | TCAGGCTCATCCCAGATACC | TTTTGCAGAGAACACACACC |
| D13Mit9 | 32.44 | GGGTTCCAGATTGAGTGGAA | TTGCCAAAGTGTCAAAATCA |
| D14Mit99 | 1.51 | TGCCATTGTGTTCAGTCTCA | TCTGCTGGGAAGACAACATG |
| D14Mit260 | 21.21 | TAGCCCAGAGACTGTCTGTTTG | TGGACTCTGGTCTACAACTATAGCA |
| D14Mit225 | 44.1 | GATATATCAAGGCTTCCTAAACACA | TCAGCATGCAGTTTAAAGTAGATG |
| D15Mit175 | 5.72 | ATAGCAACTAACAAAGACATACACACA | ACCCATTGCAGTGTAAAATTCC |
| D15Mit43 | 58.01 | GAGTTTGGTTCGGTTGTAGAGG | CTGGGTACCTCAGCTTTTGC |
| D16Mit165 | 11.17 | AAATCAGTTGGCTCTATTAGTTTGG | AATGTAAACCCTAACTAGGTCTCTCTC |
| D16Mit63 | 30.76 | CATCAGGCTGAAATGACAGTC | TGGATACGTGGCACAAAAAA |
| D16Mit152 | 48 | AGAGACCTCTGGGGTGGG | TTCAAGATAGACTATTCTGGAAAAAGC |
| D17Mit113 | 2.22 | TCTGTCTCCTCCGTACTGGG | GTCAATAAGTTCAATCACTGAACACA |
| D17Mit238 | 30.64 | TACTCCTTCCTCAACACAATACTAATT | AATGTGCTCATGCATACATGC |
| D17Mit155 | 50.71 | TGAGAAGGTTGGGTTTATATATTTAGG | CGATCATTTCCTTGCAACCT |
| D18Mit22 | 8.97 | TGATGGGATGTTTCTTGGGT | CACTGGATGACACAGCCTGT |
| D18Mit187 | 32.54 | TGCTTGAAGAAAGAGATCCTACG | GACATGCATGCCTGTAACTCC |
| D19Mit59 | 0 | CTCTAACTATCCTCTGACCTTCACA | TTTTAAGCAGAACATTGAGGACC |
| D19Mit16 | 17.16 | TCTTAGGTAATCTCCCTTAGGGG | TGGTAAATGTAAAACTGAAGCATG |
| D19Mit67 | 35.25 | GAGAAAAGTTAGCATGCGTGG | TGGTAAGTAGGCTGTTAGATATGGC |

**(Supplementary table I- continued)**

**Supplementary table II**

| **Locus** | **cM** | **FW** | **RV** |
| --- | --- | --- | --- |
| D12Mit112 | 22 | CTTCAGGCCTCCCTGGTAC | TGCCTCCAAATATACTCACAAGC |
| D12Mit54 | 24 | TGGTGAAATTCACTCCTTTGG | CCCTGTGCTGGTAGGTGTG |
| D12Mit199 | 24 | CTGCCATCCAAATCTTTGCT | ATGCATGCCACATATGTACACA |
| D12Mit89 | 28 | CTCCTTGACATCCGGGAATA | CTGTTCAAGTGCCCACCC |
| D12Mit36 | 28 | CATCACACCAGGTTTAGAATTTT | AGGCACTCTTCTGACCTCCA |
| D12Mit210 | 28 | CTGATGTGAAATTCACAAAGAACC | TGGGGCCCACTCTACATTAG |
| D12Mit69 | 28 | GAAGAGAGGACATTGCACTGG | AGTTACTGAAGCATAGACCAACCC |
| D12Mit35 | 29 | CATTCAGCTTCCTTGAGTTGC | GGTTTAGGAGTGCCCAGTCA |
| D12Mit34 | 29 | GACCACCAGGGCTATTACACA | TGCCAATCTTCACTCATGTACC |
| D12Mit114 | 29 | TTGACCTTGAACTTGTGACCC | GTTTTCTCCAAATCACTGTCACC |
| D12Mit251 | 29 | ATATTTCTCCCTCCCTTTTTTCC | CTTGTGAGGCAGAGGCAAG |
| D12Mit201 | 29 | CCACTGGATGGCAACAGAC | TATGTGTTTCAAAACCACACTCG |
| D12Mit274 | 29 | CTAAAATGGAACATAGCCACTGC | TTTTCTTGACTGTTTCTGGCTG |
| D12Mit225 | 29 | GTTAGCCAAAGCCAAATGGA | CACAATAAAATAAGCAGCAACCC |
| D12Mit212 | 29 | ACAAAGCCATTAAATCCCCC | GAGATTGACCTGAATTCAAGCC |
| D12Mit213 | 29 | GCATAAGAATTCATTGCAGTCG | GAGATTGACCTGAATTCAAGCC |
| D12Mit33 | 29 | ATCTTCAAAGTGTGCCCAGG | GCTAAAGAGAGCTCTGAATGTGG |
| D12Mit52 | 32 | CCATCTTCTGGCATTTTGCT | AGACAGGAGGGTCCCAAAGT |
| D12Mit214 | 38 | TTCATGCTCCCAAAAGGG | GCCAGTCTTTGAGATCAGGC |
| D12Mit118 | 45 | CATCTTCAATAAAATGGAGATGTACA | CGCTTTCCCTTCATGTACTAGC |
